# Early social complexity influences social behaviour but not social trajectories in a cooperatively breeding cichlid fish

**DOI:** 10.1098/rsos.230740

**Published:** 2024-03-27

**Authors:** Océane La Loggia, Alastair J. Wilson, Barbara Taborsky

**Affiliations:** ^1^ Institute for Ecology and Evolution, Behavioural Ecology Division, University of Bern, Bern, Switzerland; ^2^ Centre for Ecology and Conservation, College of Life and Environmental Sciences, University of Exeter, Penryn, UK

**Keywords:** social behaviour, life-history traits, developmental plasticity, cichlids, early-life effects

## Abstract

Social competence—defined as the ability to optimize social behaviour according to available social information—can be influenced by the social environment experienced in early life. In cooperatively breeding vertebrates, the current group size influences behavioural phenotypes, but it is not known whether the group size experienced in early life influences behavioural phenotypes generally or social competence specifically. We tested whether being reared in large versus small groups for the first two months of life affects social behaviours, and associated life-history traits, in the cooperatively breeding cichlid *Neolamprologus pulcher* between the ages of four and twelve months. As we predicted, fish raised in larger and more complex groups showed higher social competence later in life. This was shown in several ways: they exhibited more, and earlier, submissive behaviour in response to aggression from a dominant conspecific, and—in comparison to fish raised in small groups—they exhibited more flexibility in the expression of submissive behaviour. By contrast, there was no evidence that early social complexity, as captured by the group size, affects aggression or exploration behaviour nor did it influence the propensity to disperse or show helping behaviour. Our results emphasize the importance of early-life social complexity for the development of social competence.

## 1. Introduction

For group-living species, early life is especially important to develop appropriate social skills to navigate their social environment. Social competence is a form of behavioural flexibility [1,2] but specifically refers to the ability to optimize social behaviour according to available social information [1,3]. Such adjustments come with small fitness benefits that add up across social interactions [4]. In many vertebrates, the social environment experienced during early life shapes aspects of later social behaviour, including social competence [5–7]. For instance, mice reared in communal nests show a higher level of allogrooming and allosniffing towards cage mates later in life compared with mice reared in single-mother nests [6]. They also take a shorter time to adopt their respective roles in a hierarchy, reducing costs from agonistic interactions associated with resolving hierarchies [8].

High social competence is likely to have substantial fitness benefits, particularly in social species where activities necessary for survival (e.g. foraging and predator defence) and mating are frequent and require a high degree of interaction with conspecifics [1,4]. For example, in a mating context, male sage-grouse (*Centrocercus urophasianus*) have higher success when they adjust their courting effort appropriately to the presence of females [9]. In the context of within-group dominance hierarchies, social competence is often reflected in the appropriate expression of submissive behaviours, which provide powerful signals to lower the costs of an agonistic interaction between group members. These costs include the risk of injury and/or eviction, as well as the energetic costs of fighting [10–12]. In cooperatively breeding societies, where dominant group members typically monopolize breeding opportunities, submission by socially competent subordinates can act as pre-emptive appeasement to avoid punishment [13].

Effects of the current group size (and/or composition) on individual behaviours, life histories and fitness have been investigated in many cooperatively breeding vertebrates [14–18]. We know that group size can matter for fitness, with larger groups promoting survival and reproductive success in meerkats (e.g. [15,16,19]). However, much less is known about the consequences of variation in early-life social environments. The social environment experienced by individuals early in life has been shown to have a lasting influence on later-life social behaviours in some cases (e.g. altering the propensity to provide ‘help’ in the form of alloparental care [20]). The effects on other aspects of behaviour (e.g. tendency to disperse [20,21]) and life-history trajectories (e.g. investment in reproduction [22–25]) have also been documented. The group size and composition are expected to matter—larger and more diverse groups provide more complex social environments [26]. Groups comprising a complex mix of breeders and helpers, with different size classes, sexes and/or defined social roles [27], are likely to result in more numerous and diverse forms of interaction than those occurring among a uniform group of individuals (e.g. in fish schools or gnu herds [28]). Intuitively, success in complex groups may, therefore, require a higher degree of social competence, but whether or not this is facilitated by early-life exposure to complex groups is unknown.

Thus far, to our knowledge, there has been a single study experimentally varying early-life group size in a cooperatively breeding vertebrate. This experiment, done in our study species *Neolamprologus pulcher*, reported that manipulating the early-life group size and complexity affected the expression of social behaviour shortly after a defined ‘social experience’ treatment phase [29]. Fish raised in large groups had higher social competence when interacting with a larger, dominant conspecific, expressed as higher levels of submission per received aggression, compared with fish raised in small groups. This translated into higher chances to retain access to the territory of the larger fish after the hierarchy between the two fish was established [29]. In fact, several other experiments using this cichlid fish species have experimentally manipulated the early social environment, but in all cases, comparisons were between small cooperatively breeding groups and ‘socially deprived’ groups comprising sibling juveniles with no dominant breeders or subordinate helpers [5,20,30]. Fish raised in socially deprived conditions showed lower levels of social competence when interacting with more dominant fish and were less likely to be tolerated in the dominant’s territory [30]. Though interesting, these studies created a situation not found in nature; *N. pulcher* certainly shows high variation in group size in the wild, but breeders are present (together with 1–25 subordinates [26]).

In this study, we sought to manipulate the early-life group size within its natural range and determine how this affects social competence, as well as helping behaviour and propensity to leave a group (disperse). Dispersal can be viewed as a key component of life history since fish must leave their natal groups to become independent breeders. We also assessed the extent to which behavioural differences are consistent (i.e. repeatable) among individuals. The question of repeatability has thus far been ignored in the concept of social competence [1,2] but could limit the potential for selection. Assuming any single social interaction has only a small effect on fitness but that these accumulate over many interactions during life [4], then any substantive advantage to social competence requires the ability to consistently behave appropriately. Our experimental approach was to manipulate rearing groups experienced by individuals, so they differed in size and social complexity. Briefly, for the first 60 days of life, fish were raised in either large groups (a dominant breeding pair and eight subordinates of mixed sex and variable size) or small groups (a dominant breeding pair with just one small subordinate helper). Following this ‘experience phase’, fish were assayed for behavioural and life history traits at multiple subsequent timepoints using a suite of tests developed in our laboratory [20,29,31] and informed by in-depth knowledge of *N. pulcher* behaviour in its natural environment [26].

Our experiment makes particular use of the fact that *N. pulcher* have a strict linear, size-based hierarchy. This has two important consequences. First, it means that the larger rearing groups containing subordinates of variable size (and hence, rank) are more socially complex [26,27]. Second, it allows us to define expectations for socially competent behaviour in the context of interactions between a focal experimental subject and a larger (and hence, higher ranking) conspecific. Specifically, more competent individuals should show stronger (and/or faster) increases in submissive behaviour when subjected to aggression from higher-ranking fish. They should also increase submission more rapidly as the intensity of aggression received increases (e.g. if a conspecific rams or bites them rather than simply performing a threat display). We assessed this by observing focal fish interacting with larger individuals (the hierarchy test described in §2.4). Conversely, a socially competent individual should flexibly increase aggressive behaviours to assert dominance if challenged by a smaller (and therefore, lower ranking) conspecific. We assessed this by observing the behaviour of focal fish towards video-based stimuli of smaller fish displaying aggression (the *aggression test* described in §2.5). In both testing paradigms, we predicted that fish raised in larger, more complex groups will have greater social competence later in life [29], which should be reflected in both average behaviour (e.g. large-group fish will show more submission towards a higher ranking conspecific on average) and repeatability (e.g. large-group fish consistently respond appropriately).

Our primary aim is to test the hypothesized influence of early-life social environment on later social competence. Furthermore, earlier work on *N. pulcher* reared in the presence or absence of older group members indicates an association between social competence acquired in early life and adult life-history strategy. Fish raised with adults being present had higher social competence, but in addition, when they were subordinate adults, they showed lower dispersal and helping effort relative to fish raised in a socially deprived environment [20]. Based on this earlier study, we, therefore, also assayed dispersal and helping behaviour in our focal fish to see if similar patterns were detected using a more ecologically relevant manipulation by varying group size. Our prediction, based on prior empirical results, is that individuals raised in large groups will help less and choose to stay in a group more when given the opportunity to disperse.

## 2. Methods

### 2.1. Model species

The East African cichlid *N. pulcher* has proven to be a highly suitable model species in the study of the effects of the early social environment on social behaviour and life-history trajectories [5,20,25,26,30,32]. These fish breed cooperatively, with groups being organized in linear size-based hierarchies [33]. In nature, social groups are typically composed of a dominant breeding pair and subordinate helpers of various sizes and sexes [26,27]. Subordinate helpers can be related or unrelated to the breeders [34]. Helping behaviour includes alloparental care, territory defence and territory maintenance [26,35]. As cooperative breeders, they are involved in many different social interactions between group members every day and, therefore, should benefit from the early acquisition of social competence [30].

### 2.2. Rearing treatments

**Figure 1.**
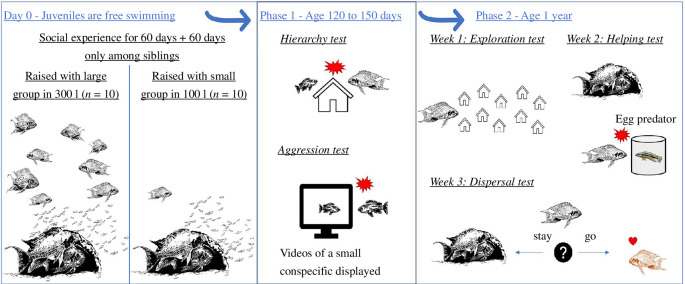
Timeline of the experiment.

We used a laboratory-bred *N. pulcher* population as the parental generation for the experimental fish, which was derived from wild-caught fish from Kasakalawe Point, Mpulungu, Zambia. We reared juvenile *N. pulcher* in the laboratory in two different early social environments: (i) small groups were composed of three adult individuals, two breeders (5.0–7.0 cm standard length (SL)) and one small helper (1.5–2.5 cm SL); and (ii) large groups were composed of 10 adult individuals, two breeders (5.0–7.0 cm SL), four large helpers (two males and two females, 4.0–4.7 cm), two medium-sized helpers (2.6–3.5 cm) and two small helpers of unknown sex (1.5–2.5 cm). Breeders and helpers were unrelated. The juveniles used for the later experiments were in most cases the offspring of the breeders of each group, although reproduction was sometimes shared among females in the large groups, so the clutches used for this experiment may have been laid by either the dominant female or one of the large female helpers. The juveniles stayed for 60 days after they were free swimming (i.e. day 0) in the group tanks for the ‘social experience phase’ (figure 1).

At day 60, the 21 broods were transferred to 50 l tanks (11 broods from small groups and 10 broods from large groups), where they were kept only among their siblings for another 60 days (‘neutral phase’). We discarded all clutches with less than four young at day 60. Discarded clutches were placed in aggregation tanks, where they grew to become a later part of our stock population. We kept sibling groups of up to 10 individuals in 50 l tanks (on average eight individuals per tank). The remaining siblings were placed together in a 200 l aggregation tank and raised for future use in other studies; they were not used in this experiment anymore. The neutral phase is important to ensure that any behavioural differences between treatments measured in the later behavioural tests are not a direct effect of the different conditions during the early-experience phase but reflect long-term developmental plastic effects.

### 2.3. Experimental phase 1

At the age of 120 days, we started the first set of tests. We selected two focal fish (average size of 2.2 ± 0.19 cm SL) per sibling group and marked them with a unique elastomer colour tag [36]. We excluded the biggest and the smallest fish, thereby excluding the fish at both ends of the size-based hierarchy to avoid differences in behaviour merely owing to their extreme rank. Furthermore, fish below 1.9 cm SL were too small to be individually marked and were, therefore, also not used. Among the remaining fish, we selected the two focal fish randomly from each sibling group.

We exposed the fish to two social challenges, a ‘hierarchy test’ and an ‘aggression test’. Both were repeated three times per fish, with hierarchy tests taking place on days 120, 127 and 150 after free swimming (figure 1). Aggression tests were carried out on the day after each hierarchy test (i.e. on days 121, 128 and 151). We tested 20 fish from the large-group treatment and 20 fish from the small-group treatment. Owing to some technical difficulties and naturally occurring mortality, some individuals could not undergo all three tests for each behavioural trait. Forty fish underwent the first test, 37 the second and 26 all the three tests. The sample size was always balanced across treatments. In experimental phase 1, the fish were not yet sexually mature, so we did not consider their sex in the statistical analysis.

### 2.4. Hierarchy test

For measuring submission, we let our focal fish interact with a larger dominant conspecific, a situation where the appropriate behaviour is to show submission in order to retain access to a territory and its resources. We introduced a focal fish in a 20 l tank equipped with a shelter as the centre of a territory and allowed it to acclimatize and claim the shelter overnight [29]. The following day we added an ‘intruder’ in the 20 l tank, which was 4–5 mm bigger than the focal fish (about 25% larger). We used a different intruder for each trial. The size of the intruder was chosen to ensure its dominance over the focal fish. We immediately recorded all aggressive and submissive displays by the focal fish and intruder for the next 20 min. We distinguished three categories of aggressive displays: fin spreads (low intensity), restrained aggression (i.e. without physical contact and medium intensity) and overt aggression (i.e. with physical contact and high intensity) [37]. We recorded the duration (in seconds) spent by the focal fish performing submissive displays, the duration of all aggressive behaviours by the intruder towards the focal fish and the latency of the focal fish to show submission for the first time. For each observed submission event, we noted the category of received aggression to which it was a response. It is worth noting that the fish did not interact non-stop during our trials, as fish sometimes swam alone in the tank or were hiding in the shelter. Four hours after the release of the intruder, we observed both fish for 10 min to establish the acceptance status of the focal fish by the larger intruder. ‘Fully accepted’ focal individuals had access to the shelter or at least to its close vicinity (<1 body length) as well as everywhere else in the tank, and the dominant did not show aggression towards the focal fish. ‘Accepted’ focal fish could swim everywhere in the tank except in the close vicinity of the shelter. The dominant was only aggressive when the focal fish tried to access the shelter. 'Fully accepted' and 'accepted' conditions were merged for the analysis under 'accepted'. ‘Evicted’ focal fish were restricted to less than a third of the tank that was the furthest away from the shelter, and they received aggression from the dominant when being close to them. In two cases, the focal fish evicted the intruder. No fish were injured in the hierarchy test. All behavioural recordings of this and the subsequent behavioural tests were done with a Sony Handycam HDR-PJ260 and coded with the software BORIS [38].

### 2.5. Aggression test

To compare aggression between treatments, we exposed our focal fish to videos of a smaller conspecific. In this situation, competent fish should be aggressive to assert dominance and keep ownership of the territory over a small conspecific. We varied the level of aggressive behaviour of the small conspecific displayed in the video to see if aggression was adjusted to these different situations. We presented videos of aggressive displays by conspecifics we had previously recorded through a one-way mirror, such that aggressive behaviours appear to be directed at the focal fish. Presenting these videos to the focal fish allowed us to standardize the intensity of the received aggression by a virtual intruder perceived by the focal fish. Video presentations to test for aggressive tendencies of focal fish were done in the same experimental tanks that were used for the hierarchy tests. We presented a video on a Samsung A5 screen, positioned vertically, of a smaller conspecific (about 1 cm on the screen) to the focal fish, which either displayed ‘fast approaches’ (strong aggression), ‘head-down’ display (low aggression) or swam calmly (no aggression) [37]. As video presentations are constrained to present restrained aggression (i.e. aggression with no physical contact), we classified the presented aggression as ‘strong’ or ‘low’ aggression. A video presentation lasted 10 min. On each test day, one presentation of each of the three aggressive levels was shown to a focal fish with a 1 h gap in between each presentation. Videos of the three aggressive levels were shown in a balanced order. We recorded all aggressive displays performed by the focal fish towards the videos. In this test, focal fish are larger than the fish on the video, and they already own a territory. Therefore, the appropriate (i.e. socially competent) behaviour to display in response to the videos is aggression towards the smaller virtual intruder and aggression of the focal fish should increase with the increased aggression levels shown by the virtual intruder in the videos.

### 2.6. Experimental phase 2

At 1 year of age (±2 weeks), we tested the focal fish for their propensities to show exploration, helping behaviour and dispersal, over a 3 week testing period (figure 1). We waited until the fish were 1 year old, as older fish engage more readily in defence behaviour [39] and fish only start to disperse after sexual maturity [40]. All tests were done in a 1000 l tank partitioned into different areas, depending on the task. We chose to test exploration propensity as an attempt to validate its use as a proxy for dispersal. We tested helping behaviour and dispersal because these traits were previously described as being part of a suite of life-history traits and social behaviours that were influenced by the early social environment [20]. At 1 year of age, 27 of the 40 individuals tested in the first experimental phase were still alive and were tested in ‘experimental phase 2’. The fish were now sexually mature (size between 3.5 and 5.0 cm SL) so that we could determine their sex and include it in the analysis.

### 2.7. Exploration test

The focal fish was introduced in a 100 l compartment of a 1000 l experimental tank (‘safe area’), which contained one flowerpot half as a shelter. On the first day, we let the focal fish acclimatize for 20 min in this compartment, before lifting a mesh divider between the safe area, where the fish were acclimatized, and an ‘exploration zone’. The exploration zone was a 600 l compartment equipped with 10 large shelters equally spaced, forming three lines across the tank bottom and a filter [31]. The shelters were placed with the opening facing the front of the tank. The focal fish was recorded for 25 min, and we counted the number of visited shelters in the exploration zone. On the second day, we repeated the same test twice, with 5 h break in between. Between the first and the second days, the fish stayed in the safe area overnight without physical or visual access to the exploration zone. Therefore, no acclimation time was necessary before the tests on day 2.

### 2.8. Helping test

We created a territory suitable for a group of fish within the 1000 l tank. The territory was 115 l composed of five large (11 × 6 cm (L × H)), three medium (10 × 6 cm (L × H)) and two small-sized shelters (8 × 4 cm (L × H)). We gave the focal fish 20 min to acclimatize to the new territory. Then, we introduced a pair of dominant breeder fish, where the dominant female was chosen to be at least 0.5 cm SL bigger than the focal fish and the dominant male at least 0.5 cm SL larger than the dominant female. We set up a neighbouring group of five adult *N. pulcher* of various sizes in a compartment next to the territory, separated by a transparent divider. The purpose of the neighbouring group was to simulate a more natural environment to raise the probability that the dominant accepts the focal fish as a helper [11].

During the second week, we performed three helping tests, each separated by 24 h (figure 1). We first placed an opaque partition between the neighbouring group and the group consisting of the pair and the focal fish, so that the neighbouring group did not distract the group during the helping test. Next, we presented an egg predator, *Telmatochromis vittatus*, inside a transparent plastic cylinder (figure 1). All used egg predators were between 4.0 and 5.0 cm SL. To prepare the presentations, we first surrounded the transparent cylinder with the egg predator in it by an opaque cylinder and allowed 10 min for both the egg predator and the pair and focal fish to acclimatize to the set-up. Then we lifted the opaque cylinder and immediately recorded the interaction of the pair and focal fish with the egg predator in its transparent tube for 15 min. Interactions seen in this test consisted of aggressive behaviours by the *N. pulcher* pair and focal fish towards the tube with the egg predator [41]. Since egg production cannot be standardized among pairs, we monitored the presence of eggs daily and removed eggs immediately when spotted. Thus, there were no eggs in the tank at the time of testing. The presence of eggs is not necessary for defence behaviour to occur. As the pairs were in the experimental tank for only a week prior to the test, egg production rarely occurred.

### 2.9. Dispersal test

For the dispersal test, we followed the method of Fischer *et al*. [20], which we summarize here briefly. The dispersal test was done in 1000 l tanks with a length of 2.6 m. At the onset of the dispersal test, the focal fish was still subordinate to the pair that had been introduced in the helping test (see §2.8). We chose an opposite-sex social partner for the focal fish that we placed in a compartment 80 cm away from the territory of the group consisting of the dominant pair and the focal fish (group territory). Thus, between the group territory and the compartment of the social partner, there was an empty 80 cm zone containing only sand, which the focal fish had to cross to disperse to the compartment containing the opposite-sex social partner. If the social partner was a female, we chose it to be 0.5–1.0 cm SL smaller than the focal fish, and if it was a male, it was larger by that size difference than the focal fish. All fish were allowed to habituate to the set-up for 14 days, with two dividers in place separating the three compartments: an opaque divider between the group territory and the empty zone, and a transparent divider between the empty zone and the social partner’s territory. After this habituation period, we removed the opaque divider between the group territory and the empty zone, and we lifted the transparent divider between the empty zone and the social partner’s territory by 2 cm. To access the social partner’s territory, the focal fish, thus, had to cross the empty zone and then swim through the 2 cm slit between the tank bottom and the transparent divider. The focal fish was allowed 7 days to decide to either stay as subordinate with the pair or form a new group in the opposite-sex social partner’s territory. On day 21, we recorded the position of the focal fish; if the focal fish was in the social partner’s territory, we considered it to have dispersed.

### 2.10. Statistical analysis

We analysed behavioural data collected from the behavioural tests using linear mixed effect models fitted in R 4.1.2 [42] using the ‘glmmTmb’ [43] and ‘LmerTest’ packages [44]. All models include early social treatment as a fixed effect and fish identity and family identity as random effects to account for repeated measures on individuals. Other fixed effects included are described below for each model. We present inference on fixed effects based on type III analysis of variance using Satterthwaite’s method. Post hoc tests for pairwise comparison between significant factors were run using the ‘emmeans’ package [45].

#### 2.10.1. Social competence

In the hierarchy test, we measured, and then modelled, four response variables: (i) the total duration of submissive displays performed by the focal fish, (ii) the focal fish’s latency to show the first submission, (iii) the duration of submission per duration of received aggression for each aggression category (low, medium and high intensity), and (iv) the acceptance status of the focal fish at the end of each test. (i) In the model with the duration of submission, the response variable was log-transformed to achieve normality of the residuals. We investigated the effect of the interaction between received aggression by the intruder and rearing treatment as well as the effect of the test number (i.e. whether it was the first, second or third test). (ii) Latency to the first submission was log-transformed to achieve normality of residuals. We investigated the effect of received aggression during the test, rearing treatment and the effect of the test number. (iii) The submission rate per received aggression was calculated as (the duration of submission within the category)/(the duration of received aggression within category + 1), with the "+1" in the denominator being included to avoid any instances of division by zero; then the rate was Box–Cox transformed to achieve normality of the distribution of residuals. We investigated the effect of the interaction of rearing treatment and category of received aggression as well as the test number on the rate of performed submission per received aggression. (iv) The acceptance status was a binary variable (accepted or not), so we used a generalized linear mixed model (GLMM) fitting a binomial distribution. We excluded the two focal fish from this analysis that evicted the intruder, as it was a rare event, and they showed the opposite behaviour patterns from all other focal fish, according to their role. Model assumptions were checked via visual inspection of residuals from Tukey–Anscombe plots and quantile–quantile plots. Additionally, we tested whether received aggression was influenced by the rearing treatment. We ran a linear mixed model (LMM) with the total duration of received aggression, which was log-transformed to achieve normality of the residuals, and with rearing treatment as covariate.

In the aggression test, we recorded the number of total aggressive behaviours performed by the focal fish against the videos and analysed this using GLMM with negative binomial error distribution as the data were overdispersed. The test number, level of received aggression and rearing treatment were included as fixed effects.

We estimated the repeatability (*R*) of submission and aggression using the ‘rpt’ function of the ‘rptR’ package [46]. We estimated the adjusted repeatability of submission duration and the number of aggressive behaviours for all fish and separated by treatment. Submission durations were log-transformed to follow a normal distribution. The repeatability of total aggressive behaviour was estimated with the data being square root transformed.

#### 2.10.2. Social trajectory traits

For each of the exploration, helping and dispersal tests, we scored and analysed a single response variable only. In the exploration test, we modelled the total number of visited pots using a negative binomial GLMM with fixed effects of test number, sex, rearing treatment and focal identity as a random effect. The same GLMM structure was then used to model the total number of aggressive behaviours against the egg predator from the helping test. We used negative binomial distribution as the data were overdispersed in both cases. Finally, we analysed dispersal as a binary variable: fish either dispersed or not. Since there was only one observation per focal fish and there was no detectable effect of family identity on the variance, we used a generalised linear model with binary distribution and fixed effects of rearing treatment, sex, size, exploration propensity and prospection on dispersal.

## 3. Results

### 3.1. Social competence

#### 3.1.1. Hierarchy test

**Figure 2.**
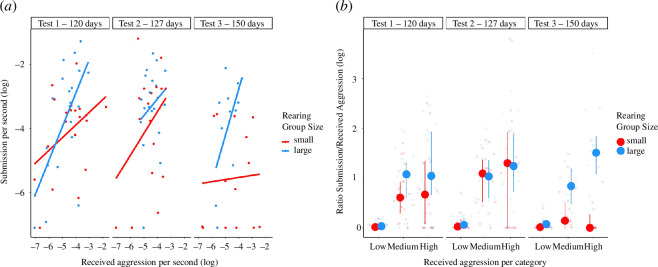
(*a*) Scatterplot representing the log of the total duration of submission per second compared to the total duration of received aggression per second during the three hierarchy tests. Blue: data points for fish reared in large groups (*n* = 20); red: data points for fish reared in small groups (*n* = 21). (*b*) Interquartile range plot representing the duration of submission for each received aggression category in the hierarchy test. The duration of submission was divided by the duration of received aggression (i.e. submission per received aggression) in each category, and the log of this ratio is shown. ‘Low’ represents the submission performed after the dominant showed fin-spread behaviour; ‘medium’ represents the submission performed after the dominant showed restrained aggression (aggression without physical contact); and ‘high’ represents the submission performed after the dominant showed overt aggression (aggression with physical contact). Blue: data points for fish reared in large groups (*n* = 20); red: data points for fish reared in small groups (*n* = 21). Medians and first and third quartiles are shown. Dots are the individual data points.

There was a significant interactive effect between the early social experience and the duration of received aggression on the duration of submission performed (figure 2*a*; LMM, *p* = 0.034; table 1*a*). Fish reared in large groups showed more submission per received aggression than fish reared in small groups (table 1*a*; figure 2*a*). They did not receive more aggression compared with fish raised in small groups (LMM, NumDF = 1, DenDF = 101, *F* = 0.74, *p* = 0.389). The test number affected the submission duration. The fish showed more submission in the second test compared with the third one (figure 2*a*; table 1*a*; LMM, *p* = 0.012). The early social environment did not affect the likelihood of being accepted in the intruder fish territory (GLMM, *χ*_1_² = 0.029, *p* = 0.866).

**Table 1.** Results of the LMMs fitting the effects on submission in the hierarchy test. ((*a*) Effects of the test numbers (1, 2 and 3), size and rearing treatment, received aggression and their interaction on the duration of submission. The duration of submission was log-transformed to achieve normality of model residuals (*n* = 100 observations, *n* = 40 individuals and *n* = 21 family groups). (*b*) Effects of rearing treatment, category of received aggression (i.e. fin spread (score of 1), restrained aggression (2) or overt aggression (3) performed by the dominant individual that triggered submission events by the subordinate individual) and their interaction, and the test number and the size on the ratio of the duration of submission per duration of received aggression in each category. The duration of submission was Box–Cox transformed to achieve normality of model residuals (*n* = 300 observations, *n* = 40 individuals, and *n* = 21 family groups). (*c*) Effects of the test number (1, 2 or 3), rearing treatment and received aggression on the latency to first submission (log-transformed) (*n* = 91 observations and *n* = 40 individuals). The post hoc results are pairwise comparisons between (*d*) the rearing treatments and test numbers from models (*a*) and (*e*) between rearing treatments compared within each category of received aggression and test number from the model (*b*). The duration of submission was Box–Cox transformed to achieve normality of model residuals; the interpretation of estimates has to take account of this data transformation, which caused the estimate signs to reverse in direction. NumDF: numerator degrees of freedom. DenDF: denominator degrees of freedom. Significant *p-*values are highlighted in bold.)

model		factor level	estimate	s.e.	numDF	denDF	*F-*value	*p-*value
(a) time spent performing submission (total)								
rearing treatment × received aggression			52.809	22.931	1	79.955	5.304	**0.024**
rearing treatment		large	0.966	0.428	1	19.569	5.096	**0.036**
received aggression			6.661	7.205		80.12	8.402	**0.005**
test number					2	86.704	4.263	**0.017**
		test 2	0.273	0.315				
		test 3	−1.032	0.465				
size			0.155	0.941	1	49.039	0.279	0.599
(b) time spent performing submission after receiving aggression								
rearing treatment × category of received aggression					2	255.16	5.099	**0.007**
		large × restrained aggression	−0.124	0.601				
		large × overt aggression	−0.191	0.061				
rearing treatment		large	0.004	0.06	1	18.88	4.243	0.053
category of received aggression					2	255.16	112.793	**<0.001**
		restrained aggression	−0.328	0.043				
		overt aggression	−0.305	0.043					
test number					2	228.11	7.167	**<0.001**
		test 2	−0.052	0.029				
		test 3	0.109	0.047				
size			−0.065	0.107	1	53.315	0.36	0.55
(c) latency to the first submission								
rearing treatment		large	−0.632	0.282	1	17.276	5.018	**0.039**
received aggression			0.216	6.026	1	72.663	0.001	0.971
test number					2	60.476	0.5	0.609
		test 2	0.051	0.258				
		test 3	0.387	0.391				
size			−0.267	0.715	1	29.438	0.14	0.711

pairwise comparisons				estimate	s.e.		T ratio	*p*‐value
(d) time spent performing submission								
small–large				−0.974	0.431		−2.261	**0.035**
test 1–test 2				−0.273	0.316		−0.864	0.664
test 1–test 3				1.032	0.478		2.161	0.083
test 2–test 3				1.305	0.457		2.858	**0.015**
(e) time spent performing submission after receiving aggression								
	low aggression							
small–large				−0.004	0.06		−0.075	0.941
	medium aggression							
small–large				0.112	0.06		1.983	0.054
	high aggression							
small–large				0.188	0.06		3.101	**0.003**
test number								
test 1–test 2				0.052	0.029		1.786	0.178
test 1–test 3				−0.109	0.482		−2.265	0.064
test 2–test 3				−0.161	0.045		−3.584	**0.001**

Overall, submission duration across the three observations in the hierarchy test was repeatable (repeatability adjusted for test number, *R* = 0.213, confidence interval (CI) = [0,0.447], *p* = 0.023). When estimated separately by early-life treatments, submission was only significantly repeatable in fish raised in large groups (large group: *R* = 0.279, CI = [0,0.606], *p* = 0.049; small group: *R* = 0.050, CI = [0,0.377], *p* = 0.357). Based on the wide and strongly overlapping CIs, we note, however, that R does not itself differ significantly between the two treatment groups.

**Figure 3.**
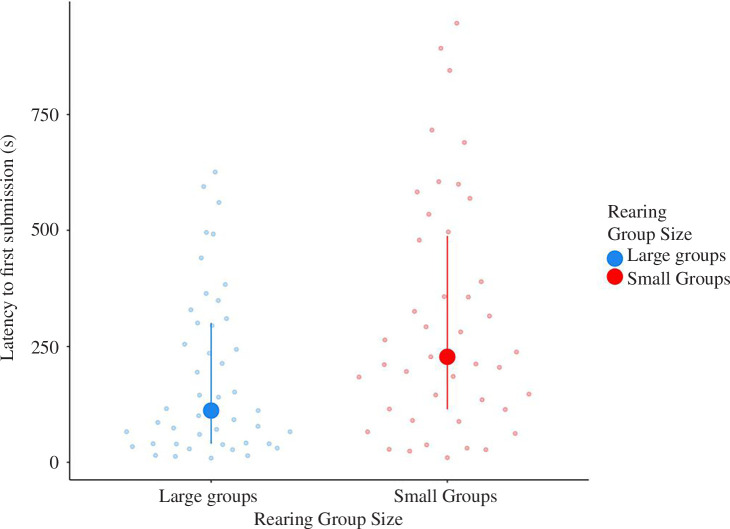
Interquartile range plot representing the latency to the first submission in the hierarchy test. Medians and first and third quartiles are shown. Dots are individual data points. Blue: data points for fish reared in large groups (*n* = 20); red: data points for fish reared in small groups (*n* = 21).

When calculating the relative duration of submission shown in response to different categories of received aggression reflecting increasing aggressive intensity (low: fin spread; intermediate: threat display; high: overt aggression), there was also a significant interaction between the rearing treatment and the category of received aggression triggering the submissive event (figure 2*b*; table 1*b*; LMM, *p* = 0.007). Post hoc analyses showed that the fish reared in large groups have a steeper response when the intensity of received aggression increases compared with fish raised in small groups (figure 2*b*; table 1b; pairwise comparisons: medium *p* = 0.030, high *p* < 0.001). This means that fish reared in large groups exhibited relatively more submission with increasing levels of received aggression. There was again an effect of the test number on submission duration per category of received aggression. Fish showed less submission per category of received aggression in the second test compared to the third test (figure 2*b*; table 1b; LMM, *p* < 0.001). Finally, fish from the large-group treatment also showed a shorter latency to show the first display of submission than fish from the small-group treatment (figure 3; table 1*c*).

#### 3.1.2. Aggression test

On average, focal fish increased aggression to more aggressive stimuli (table 2; GLMM, *p* < 0.001). There was neither a main effect of early-life treatment on the aggressiveness of the focal fish (table 2; GLMM, *p* = 0.280), nor was there any support for an interaction between treatment and stimulus aggression level. This means that large- and small-group focal fish do not differ in how they adjust their own aggression according to the level of aggression of the stimulus fish in the video. Fish reduced their aggression frequency across tests (table 2; GLMM, *p* < 0.001). Aggressive behaviour was repeatable across all observations (*R* = 0.388, s.e. = 0.073, CI = [0.231,0.519], *p* < 0.001). Estimates of repeatability for aggressive behaviour were almost identical in the subsets of data of fish raised in different early-life environments (large groups: *R* = 0.383, s.e. = 0.107, CI = [0.154,0.568], *p* < 0.001; small groups: *R* = 0.398, s.e. = 0.110, CI = [0.170,0.582], *p* < 0.001).

**Table 2.** A GLMM assuming a negative binomial distribution, to test the effects of rearing treatment, test the number and level of received aggression in the video (low, medium and high) on aggression in the aggression test (*n* = 508 trials, *n* = 38 individuals). (Significant *p-*values are highlighted in bold.)

fixed effects	factor level	estimate	s.e.	d.f.	*χ*²	*p-*value
rearing treatment	small	0.746	0.865	1	1.051	0.305
received aggression		1.361	0.124	2	105.288	**<0.001**
test number				2	32.525	**<0.001**
	test 2	0.189	0.344			
	test 3	−2.784	0.425			

### 3.2. Social trajectory traits

#### 3.2.1. Exploration test

**Figure 4.**
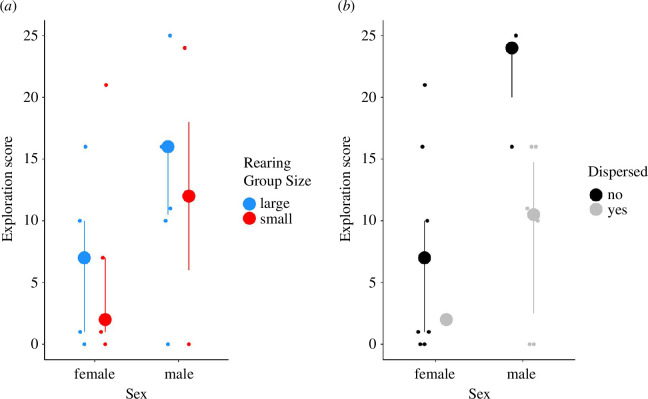
Interquartile range plot representing the exploration score (total number of pots visited) by the fish during the exploration task between (*a*) rearing group size and sex and (*b*) disperser status and sex. Blue: fish raised in large groups (*n* = 14); red: fish raised in small groups (*n* = 13); black: non-dispersers; and grey: dispersers. Medians and first and third quartiles are shown. Small dots are individual data points.

**Figure 5.**
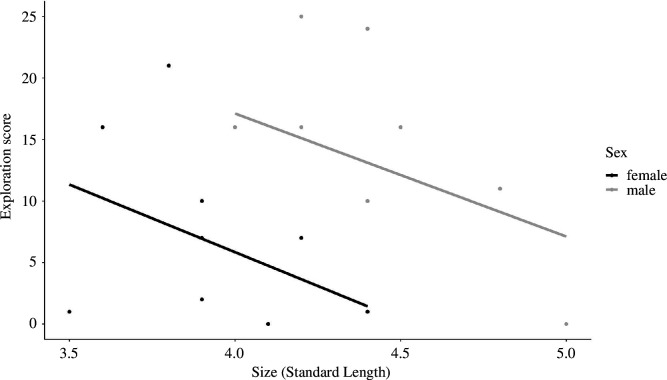
Plot representing the exploration score (total number of pots visited) in the exploration task for fish of different sizes (SL, *n* = 21). Regression lines and individual scores. Black: females; grey: males.

The early-life treatment did not affect explorative behaviour; fish showed more exploration in the second test (table 3; GLMM, *p* = 0.036). Males were more explorative than females (figure 4*a*; GLMM, *p* = 0.005; table 3), and smaller fish were more explorative than larger fish (figure 5; GLMM, *p* = 0.030).

**Table 3.** A GLMM assuming a negative binomial distribution, to test the effects of rearing treatment, test number, sex and size on the total number of visited pots in the exploration task (*n* = 63 trials, *n* = 21 individuals). (The post hoc results are pairwise comparisons between test number and sex. Significant *p-*values are highlighted in bold.)

fixed effects	factor level	estimate	s.e.	d.f.	*χ*²	*p-*value
rearing treatment	small	0.034	0.039	1	0.008	0.93
test number				2	6.618	**0.037**
	test 2	1.264	0.477			
	test 3	0.905	0.48			
sex	male	1.772	0.594	1	7.761	**0.005**
size		−1.855	0.842	1	4.604	**0.032**

comparisons			estimate	s.e.	d.f.	*p-*value
test number						
test 1—test 2			−1.264	0.477	54	**0.028**
test 1—test 3			−0.905	0.48	54	0.153
test 2—test 3			0.359	0.444	54	0.699
sex						
female–male			−1.77	0.594	54	**0.004**

#### 3.2.2. Helping test

**Figure 6.**
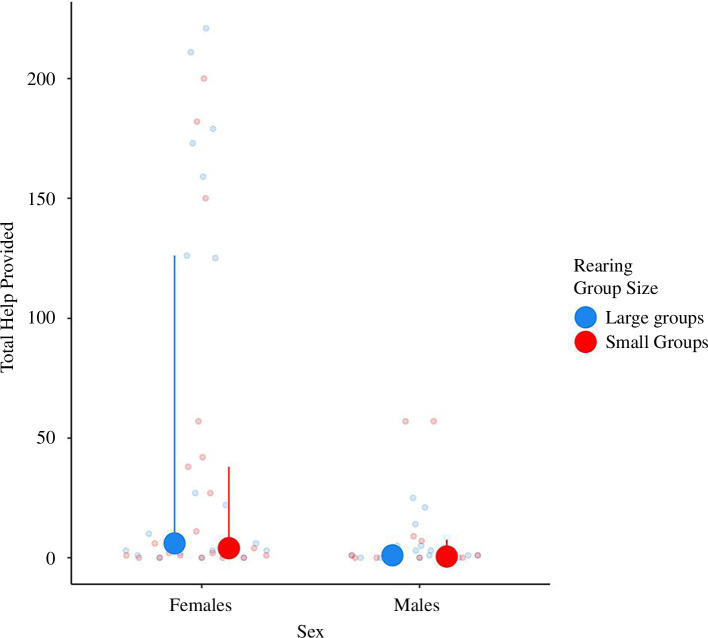
Interquartile range plot representing the total number of aggressions performed by the fish towards an egg predator intruder during the helping task. Medians and first and third quartiles are shown. Dots are individual data points. Blue: data points for fish reared in large groups (*n* = 14); red: data points for fish reared in small groups (*n* =13).

There was no effect of the early-life treatment on the amount of helping behaviour (GLMM, *p* = 0.265; table 4). Females showed more helping behaviour than males (figure 6; GLMM, *p* = 0.009; table 4).

**Table 4.** A GLMM assuming a negative binomial distribution, on the effects of rearing treatment, test number and sex on the total number of helping behaviour performed by the focal individual in the helping task (*n* = 74 trials, *n* = 27 individuals). (Significant *p-*values are highlighted in bold.)

fixed effects	factor level	estimate	s.e.	d.f.	*χ*²	*p-*value
rearing treatment	large	0.862	0.777	1	1.243	0.265
test number				2	3.957	0.138
	test 2	0.532	0.289			
	test 3	0.078	0.322			
sex	male	−2.252	0.839	1	6.818	**0.009**

#### 3.2.3. Dispersal test

The early-life treatment did not influence the dispersal behaviour of the fish (GLMM, *p* = 0.334; table 5a) nor did it influence the time the fish spent prospecting at the edge of the dominant’s territory (GLMM, *p* = 0.786; table 5b). Time spent prospecting before the dispersal decision did not predict dispersal (GLMM, *p* = 0.085; table 5a). However, the exploration score from the exploration test predicted the level of dispersal; fish showing a higher exploration score were less likely to disperse (GLMM, *p* = 0.014; table 5a; figure 4*b*). Males were more likely to disperse than females (GLMM, *p* = 0.019; table 5a). We had nine fish dispersing out of 27 in the experiment, from these nine fish, one was a female, and the others were all males.

**Table 5.** (*a*) A GLMM assuming a binomial distribution, to test for the effects of rearing treatment, sex and exploitation score (total number of pots visited in the exploration task) on dispersal (binary variable 0 or 1) (*n* = 21 trials, *n* = 21 individuals). (*b*) LMM to test for effects of rearing treatment, sex and size on prospecting behaviour, described as the time spent near the door dividing the dominant’s territory from the potential mate’s territory (*n* = 44 trials, *n* = 24 individuals). (Significant *p-*values are highlighted in bold.)

fixed effects	factor level	estimate	s.e.	d.f.	*χ*²	*p*-value
(a) dispersal						
rearing treatment	small	2.635	3.188	1	0.969	0.324
sex	male	13.534	9.312	1	12.735	**0.0004**
exploration score		−0.645	0.473	1	7.705	**0.005**
prospecting		0.022	0.041	1	0.341	0.559

fixed effect	factor level	estimate	s.e.	NumDF	DenDF	*F* value	*p*-value
(b) prospecting							
rearing treatment	small	−0.273	0.653	1	10.519	0.175	0.685

## 4. Discussion

Our study investigated the effect of early social group size and complexity on social competence and social trajectories. First, our results demonstrate the importance of a larger early social group size, which in our study species is related to greater social complexity, for the development of better social competence. Fish that experienced large groups early in life containing a mix of adults and juveniles of different sexes, sizes and social ranks showed a steeper increase in submission relative to increasing received aggression. Fish raised in large groups increased their submission relative to increasing received aggression both in frequency and intensity, and they adopted the appropriate subordinate behaviour faster in a social contest with a larger conspecific. The amount of received aggression did not differ between fish raised in large and small groups. In summary, fish raised in large groups showed more appropriate *and* more flexible behavioural responses when interacting with a dominant fish. However, there was no influence of the early social environment on aggressive behaviour towards videos of smaller conspecifics. The focal fish showed consistent aggressive behaviour towards the videos, and they increased their aggression when the video showed a more aggressive individual. This shows that the fish recognized the behaviours on the videos, and it further suggests that our fish from both early social treatments showed appropriate responses when competing against a smaller conspecific. There was also no evidence for the effects of the early social environment on explorative, helping and dispersal behaviour. Taken together, these results suggest that varying early social group size and complexity has long-term effects on submissive behaviour but does not induce divergence in social and life-history trajectories, contrary to what has been reported previously in fishes raised in natural versus socially deprived social conditions [20].

The ability to flexibly adjust one’s level of appropriate behaviour towards the behaviour of social partners during interactions is one indicator of social competence in animals [1]. In *N. pulcher*, an expression of higher social competence is to show a stronger response of submissive behaviour towards received aggression by a dominant fish [30]. In this species, the hierarchy is linear and size-based [33]. Thus, submission is the appropriate behaviour to show when interacting with a larger conspecific, which in *N. pulcher* is typically dominant over smaller individuals because of its size advantage. Generally, submission helps avoid escalated fights and injuries [12] and enables the formation of a stable dominance hierarchy [47]. In *N. pulcher*, submissive behaviour is a very important mechanism to regulate aggression in the group; it can even be expressed to achieve pre-emptive appeasement in order to avoid punishment by dominants in large and small groups [13,48,49]. Alternatively, one could argue that those fish showing lower submission per received aggression are more efficient in solving conflicts. However, this is unlikely because previous studies showed several benefits for fish showing high submission per received aggression, including reduced duration of conflicts [5], being tolerated closer to a shelter [30] receiving less aggression and being more often accepted by dominant breeders [20,30] and, accordingly, being less often evicted from a territory [30]. One may also argue that too much submission is counterproductive and may lead to higher eviction rates from the group territory as was shown in a pharmacological study applying isotocin to *N. pulcher* [50]. However, in unmanipulated fish, there was either no difference in eviction rates (this study) or fish were less often evicted [30] when showing more submission per received aggression.

Another alternative possibility for the observed response difference between fish raised in small and large groups might be that they differed in some other aspect; for instance, size, growth or body condition. However, in our study, fish did not differ in size between social treatments (LMM, d.f. = 1, estimate = − 0.013, *p* = 0.87; measured at 4 months of age). Moreover, previously, *N. pulcher* raised in small or large group did not differ in specific growth rate [29], and fish raised with or without adults did not differ in their body condition [5]. Therefore, it seems unlikely that the observed differences in responding to received aggression can be explained by divergence in size, growth or body condition induced by their early environment, and we believe that the experimentally induced group size effect most likely reflects a difference in social competence.

To gain the accumulated benefits of social competence during the multitude of interactions that group-living animals have every day, individuals need to consistently be able to adjust their responses appropriately to the current situation [1]. Here, we found that fish raised in large groups showed repeatable durations of submissive behaviour across the three observations but also responded appropriately (on average) to varying levels of aggression received. Our current data are not directly informative on rearing treatment differences for the repeatability (or consistency) of this plastic response by individuals to aggression received, because we lacked the power to properly test the effect of the rearing group size on repeatability. Nonetheless, we propose that this is an important question that has thus far been neglected in the study of social competence. Interestingly, our estimate of repeatability for submissive behaviour was lower (and non-significant) for fish raised in small groups, suggesting that early-life experience can influence the structure of among-individual differences. However, we also acknowledge that the repeatability estimates have wide CIs and did not differ significantly between treatments, so a more targeted experimental design may be required to formally investigate this.

Previous findings suggest that the early social environment triggers a life-long divergence of social and life history trajectories in *N. pulcher* [20,25] with a divergence between two social phenotypes, one being socially competent and philopatric but with low helping propensity and reproductive investment in egg size and number, and a second type with the opposite profile. When measuring a suite of social and non-social behavioural and life-history traits, we found long-lasting effects over five months after the early social experience on the propensity to show submission but not on other social and non-social traits. Thus, there was no evidence of an overarching behavioural phenotype induced by the early social environment. This may be explained by the major difference between previous and our studies. While we contrasted large and small natural group sizes, previous *N. pulcher* studies compared group-living versus socially deprived (brood mates only) rearing settings [20,25]. A recent long-term field study on *N. pulcher* found that group size did not influence the likelihood of dispersal [40], suggesting that the rearing conditions of the previous studies (socially deprived versus natural social groups) may have represented a stronger contrast of the social environments, thus affecting more traits than the comparison of two different natural group sizes as done in our study.

Only a few fish in the dispersal test dispersed to a neighbouring compartment containing a potential mate for independent breeding. These were mostly males. A previous study investigating social trajectories in *N. pulcher* used 3-year-old fish, but owing to the advanced age, aggression was high in males, and group integration followed by dispersal could only be investigated in females [20]. Therefore, here, we used younger fish of 1 year of age. While with this approach, we were able to study dispersal in both sexes, the younger age may also explain the generally low dispersal propensity in our experiment. It is likely that some of our fish were still too small to disperse for independent breeding. However, both sexes will improve their social rank by dispersing, as they will occupy either the first or second rank in the hierarchy of a small social unit. Males were the more explorative sex and had a higher propensity to disperse than females, which is in line with natural dispersal patterns in this species [40] and findings from the laboratory [31]. It is also the general pattern in mammals and other fish species [51–53]. For example, like in *N. pulcher*, in meerkats (*Suricata suricatta*), females display higher levels of help compared with males and show higher levels of philopatry [54]. Moreover, in our study, males showed lower levels of help than females, thus validating previous findings [31]. Sex is an important determinant of life-history strategies in vertebrates [31,54]. However, there is still behavioural variation within sex that can be explained by early-life experience. In coyotes (*Canis latrans*), for instance, individuals of both sexes differ in their dispersal decisions depending on their environment in early life [21].

We had expected that explorative behaviour would be positively related to dispersal propensities as this link has been widely reported in other species [55,56]. Against our expectations, more explorative individuals actually dispersed less. Exploration is susceptible to vary with time. For instance, in root voles (*Microtus oeconomus*)*,* behavioural differences, including exploration differences, between disperser and resident are only temporary [57]. Prospecting before a dispersal decision did not impact whether a fish dispersed or not, so it could be that our measure of exploration was disconnected from the dispersal decision. In addition, exploration can also be the result of different environmental pressures. In European hares (*Lepus europaeus*)*,* exploration is probably triggered by predation rather than by dispersal [58]. In line with these findings, it is possible that exploring novel shelters adjacent to the home territory (like in the exploration task) and dispersing to settle with a mate in a new territory (like in the dispersal task) represent two entirely different and unrelated ecological contexts for *N. pulcher*.

Animals living in social groups engage in many social interactions with conspecifics every day. For group-living individuals, being able to competently behave in each of the many social interactions occurring in social groups will bring higher fitness benefits. Such benefits could include, for example, avoiding injuries or saving energy expenditures during contests [10]. Here, we demonstrate that larger groups produce more socially competent offspring. Individuals with the appropriate social skills are more likely and faster to find their appropriate social role in a hierarchy [8] and more likely to be accepted and stay in social groups [30]. If social competence is transmitted environmentally or genetically across generations [4], this can contribute to increased group sizes and, therefore, to the enhanced production of socially competent individuals. This can generate a positive feedback loop based on individual-to-society feedback [59], enhancing sociality and promoting the formation of even larger groups of individuals [4]. Living in large groups can yield fitness benefits such as increased survival [16,18], better territory quality [14] and increased reproductive success for breeders [14,15,17,60]. Living in larger groups can also promote cognitive development as shown in cooperatively breeding Australian magpies (*Gymnorhina tibicen*). Individuals born and living in larger groups performed better in a series of cognitive tasks and benefitted from the facilitated transmission of information [61,62], and task performance was positively related to indicators of reproductive success [61], suggesting potential fitness benefits of group living via the acquisition of better cognitive skills.

In conclusion, our findings support the large body of the literature showing that developmental plasticity has a key role in the expression of social behaviour and, more specifically, in the expression of social competence. We showed that increasing the early social group size and complexity enhances social competence in a cooperative breeder. We stressed that social competence is likely to benefit an individual through cumulated fitness benefits over numerous social interactions; therefore, consistency of social competence is crucial to rendering this social ability beneficial. However, we did not find differences in other social traits that resulted from developmental plasticity, suggesting that the group size and complexity do not shape life-long life-history trajectories in *N. pulcher*. Taken together our results highlight the importance of the early social environment in shaping social competence.

## Data Availability

Data is available as supplementary material [63].
